# A novel therapeutic management for diabetes patients with chronic limb-threatening ischemia: comparison of autologous bone marrow mononuclear cells versus allogenic Wharton jelly-derived mesenchymal stem cells

**DOI:** 10.1186/s13287-023-03427-z

**Published:** 2023-08-25

**Authors:** Martha L. Arango-Rodríguez, Ligia C. Mateus, Claudia L. Sossa, Silvia M. Becerra-Bayona, Víctor Alfonso Solarte-David, Miguel Enrique Ochoa Vera, Lady T. Giratá Viviescas, Ana M. Vera Berrio, Sergio Eduardo Serrano, Oliverio Vargas, Andrés Catalá Isla, Alape Benitez, Germán Rangel

**Affiliations:** 1 Banco Multitejidos y Centro de Terapias Avanzadas, Clínica FOSCAL Internacional, 681004 Floridablanca, Colombia; 2 Fundación Oftalmológica de Santander Carlos Ardila Lulle, 681004 Floridablanca, Colombia; 3 Programa para el Tratamiento y Estudio de Enfermedades Hematológicas y Oncológicas de Santander (PROTEHOS), 681004153 Floridablanca, Colombia; 4 Facultad de Ciencias de la Salud, Universidad Autónoma de Bucaramanga - UNAB, 681003 Bucaramanga, Colombia; 5 Facultad de Ingeniería, Universidad Autónoma de Bucaramanga - UNAB, 680003 Bucaramanga, Colombia

**Keywords:** Peripheral arterial disease, Chronic limb-threatening ischemia, Cell therapy, Autologous bone marrow mononuclear cells and allogenic Wharton jelly-derived mesenchymal stem cells

## Abstract

**Background:**

Chronic limb-threatening ischemia (CLTI) represents the final stage of peripheral arterial disease. Approximately one-third of patients with CLTI are not eligible for conventional surgical treatments. Furthermore, patients with advanced stage of CLTI are prone to amputation and death. Thus, an effective therapeutic strategy is urgently needed. In this context, autologous bone marrow mononuclear cell (auto-BM-MNC) and allogeneic mesenchymal stem cells represent a promising therapeutic approach for treating CLTI. In this study, we compared the safety and beneficial therapeutic effect of auto-BM-MNC versus allogeneic Wharton jelly-derived mesenchymal stem cells (allo-WJ-MSCs) in diabetic patients with CLTI.

**Methods:**

We performed a randomized, prospective, double-blind and controlled pilot study. Twenty-four diabetic patients in the advanced stage of CLTI (4 or 5 in Rutherford’s classification) and a transcutaneous oxygen pressure (TcPO_2_) below 30 mmHg were randomized to receive 15 injections of (i) auto-BM-MNC (7.197 × 10^6^ ± 2.984 × 10^6^ cells/mL) (*n* = 7), (ii) allo-WJ-MSCs (1.333 × 10^6^ cells/mL) (*n* = 7) or (iii) placebo solution (1 mL) (*n* = 10), which were administered into the periadventitial layer of the arterial walls under eco-Doppler guidance. The follow-up visits were at months 1, 3, 6, and 12 to evaluate the following parameters: (i) Rutherford’s classification, (ii) TcPO_2_, (iii) percentage of wound closure, (iv) pain, (v) pain-free walking distance, (vi) revascularization and limb-survival proportion, and (vii) life quality (EQ-5D questionnaire).

**Results:**

No adverse events were reported. Patients with CLTI who received auto-BM-MNC and allo-WJ-MSCs presented an improvement in Rutherford’s classification, a significant increase in TcPO_2_ values‬, a reduction in the lesion size in a shorter time, a decrease in the pain score and an increase in the pain-free walking distance, in comparison with the placebo group. In addition, the participants treated with auto-BM-MNC and allo-WJ-MSCs kept their limbs during the follow-up period, unlike the placebo group, which had a marked increase in amputation.

**Conclusions:**

Our results showed that patients with CLTI treated with auto-BM-MNC and allo-WJ-MSCs conserved 100% of their limb during 12 months of the follow-up compared to the placebo group, where 60% of participants underwent limb amputation in different times. Furthermore, we observed a faster improvement in the allo-WJ-MSC group, unlike the auto-BM-MNC group.

*Trial registration* This study was retrospectively registered at ClinicalTrials.gov (NCT05631444).

**Supplementary Information:**

The online version contains supplementary material available at 10.1186/s13287-023-03427-z.

## Introduction

Chronic limb-threatening ischemia (CLTI) constitutes the most severe condition of peripheral artery disease (PAD) [1]. Patients with diabetes mellitus have four times higher risk of developing CLTI [2]. Similarly, diabetic patients have a fivefold higher amputation risk, worse PAD outcomes, more foot pain, higher number of non-healing ulcers, and higher mortality rates than non-diabetic patients with a similar smoking history, ischemic heart disease, and hypercholesterolemia [3].

PAD and diabetes are main risk factors for lower limb amputation [4]. In spite of significant advances in revascularization methods, many patients with CLTI are still considered unsuited for these procedures, among others, due to previous operations or general inoperability. Thus, these patients are treated with conservative limb therapies, e.g., the best medical treatment of risk factors plus pain medication and wound treatment [5]; however, many end up with amputations. For instance, a cohort study showed that once the patients have developed CLTI, even with revascularization (peripheral angioplasty) or bypass graft, 8.2–21.5% of patients underwent substantial amputation within six years, 14.6% of the patients needed secondary revascularization after the first surgery, and 4.9% of these patients were not eligible for revascularization [6].

Therefore, new biological revascularization options to improve CLTI are under study, among which cell-based therapy offers the greatest hope for these patients. These therapies have provided new prospects for patients without conventional, open, or endovascular therapeutic options for two decades by potentially enabling neo-angiogenesis.

The rationale behind using cell-based therapy as a treatment for ischemic cardiovascular disease was motivated by the discovery that human blood contained progenitor cells that mobilize to ischemic tissues and augmented angiogenesis [7, 8]. As a result, the first generation of cell-based therapy trials was conducted using bone marrow mononuclear cells (BM-MNC), a direct bone marrow isolated that contains different cell types, mainly from the hematopoietic line stem cells and endothelial progenitor cells (EPCs).

These EPCs were initially thought to promote angiogenesis by forming new vessels as they actively homed to ischemic areas after injection. They contribute to vascular regeneration by differentiating into mature endothelial cells (EC), forming a structural component of capillaries and secreting angiogenic factors. However, several studies have demonstrated that diabetic patients showed a reduced number of EPCs, that are dysfunctional, and the cultured EPCs presented profound functional impairment [9–11]. Together, these data suggest that the reduction and dysfunction of EPCs in diabetes mirror an insufficient endogenous regenerative capacity, and favor the development of vascular complications that lead to extensive vascular damage in these patients.

Recently, a number of researchers have conducted clinical studies on treating CLTI with other stem cells. Indeed, a recent study by Khodayari et al. reveals that endometrial tissue can be considered a suitable candidate for isolating new safe, effective, and feasible multipotent stem cells (endometrial-derived stem cells (EnSCs)) for limb regeneration. EnSCs can generate diverse types of cells, essential for limb reconstruction, including endothelial cells, smooth muscle cells, muscle cells, and even peripheral nervous system populations (Khodayari, 2022 #70).

On the other hand, the most common stem cells include CD34^+^ bone marrow cells and mesenchymal stem cells (MSCs) derived from different sources (bone marrow, adipose tissue, and, recently, umbilical cord). These therapies have gained interest as a potential treatment to enhance vascularization either through a direct effect of the administered cells on the vasculature or by secretion of pro-angiogenic factors and modulation of the local immune response, which prevent amputation in patients with CLTI [12]. Nevertheless, it is still unclear whether autologous BM-MNC or allogeneic MSCs are more effective in diabetic patients with CLTI.

In our present work, we conducted a pilot study to determine the safety and beneficial therapeutic effect of auto-BM-MNC and allogeneic Wharton jelly-derived mesenchymal stem cells (allo-WJ-MSCs) as treatments for diabetic patients with CLTI. Briefly, we compared the used of one dose of auto-BM-MNC, one dose of allo-WJ-MSCs, or one dose of placebo solution (saline solution with 2% of autologous serum), which were injected into the periadventitial layer of the arterial walls under eco-Doppler guidance. In addition, the (i) Rutherford’s classification, (ii) TcPO_2_, (iii) percentage of wound closure, (iv) pain, (v) pain-free walking distance, (vi) revascularization and limb-survival proportion, and (vii) the clinical outcome scale (EQ-5D questionnaire) were evaluated during the follow-up (12 months).

## Material and Methods

### Study design

We performed a randomized, prospective, double-blind, controlled, and parallel-group pilot study at the Fundación Oftalmológica de Santander (FOSCAL-(http://www.foscal.com.co/servicios/)) (Bucaramanga-Colombia) to assess the safety and beneficial therapeutic effect of one dose of auto-BM-MNC and allo-WJ-MSCs, which were injected into the periadventitial layer of the arterial walls on diabetic patients with CLTI. The study was conducted following Good Clinical Practice guidelines and the Declaration of Helsinki. The Research Ethics Committee approved all protocols at FOSCAL, Colombia (Act. No. 17/May 26th, 2017), and the study was registered at ClinicalTrials.gov (NCT05631444). Before auto-BM-MNC or allo-WJ-MSC isolation, written informed consent was obtained from bone marrow donors or umbilical cord donors as well as study participants.

### Participants

The target population included 51–85 years old diabetic patients of both sexes with a basal Rutherford’s classification stage of 3 to 5, recruited between January 2019 and October 2021 at FOSCAL. All participants met the inclusion criteria described in Table 1. However, most of the study participants did not have appropriate metabolic control of diabetes (hemoglobin A1c mean total 7.32 ± 0.94) and lipid levels (total cholesterol mean total 139.2 ± 33.36, triglycerides mean total 190.4 ± 148, high-density lipoprotein mean total 38.25 ± 13.37, and low-density lipoprotein mean total 73.76 ± 27.80) before and during the study. In addition, the demographic and baseline characteristics of participants are described in Tables 2 and 3, respectively.

**Table 1:** Inclusion and exclusion criteria

Inclusion criteria	Exclusion criteria
Adult male or female, 40 years of age or over (until 86 years old)	Participants that do not sign the informed consent
TcPO_2_ ≤ 30 mmHg	Presence of osteomyelitis
Diagnosis of diabetes	Hemodynamic instability (MAP < 65 mmHg or vasopressor requirement)
Patients with signs of critical ischemia such as:	Any acute systemic infectious disease process
Ulcer that does not heal	
Necrosis or loss of tissue	
Pain at rest	
Intermittent claudication	
Basal Rutherford classification stage 3 to 5	Severe sepsis
Non-revascularized patients due to comorbidities and/or anatomy	Uncontrolled coagulopathy
Patients that despite revascularization (vascular surgery) have adequate distal beds to perfuse the limb	Condition of cancer
Ankle/brachial index less than 0.4	Use of immunosuppressive or cytotoxic drugs
Stenosis or occlusion of the infrapatellar arteries	Alterations of the bone marrow that do not allow the adequate extraction of the components to be used as: acute leukemia, chronic leukemia, marrow aplasia, myelodysplastic syndrome, and myelophthisis
	Contraindication of sedation for bone marrow aspirate
	Patients who have suffered in a period < six months of myocardial infarction, disease cerebrovascular or coronary intervention
	Patients with liver failure indicated by serum transaminases (aspartate aminotransferase and alanine aminotransferase), with values twice the normal limit
	Any acute or chronic contagious disease including hepatitis B, hepatitis C, and HIV
	Any other comorbidity that the treating vascular surgeon considers as a contraindication to cell treatments

**Table 2:** Demographic characteristics of participants

	Group		
	Placebo	Auto-BM-MNC	Allo-WJ-MSCs
*Sex*			
Male, *n* (%)	5 (50)	7 (100)	5 (71)
Female, *n* (%)	5 (50)	–	2 (29)
*Age (years)*			
18–50 years, *n* (%)	–	–	–
51–85 years, *n* (%)	9 (100)	7 (100)	6 (100)
Mean ± SD	73.11 ± 10.56	72.43 ± 5.06	78.33 ± 5.85
Median	77	71	77.50
Min/max	58/84	67/80	70/85

*Auto-BM-MNC* autologous bone marrow mononuclear cells, *allo-WJ-MSCs* allogenic Wharton jelly-derived mesenchymal stem cells and *SD* standard deviation

**Table 3:** Baseline characteristics of participants

	Group		
	Placebo	Auto-BM-MNC	Allo-WJ-MSCs
Glycated hemoglobin A_1c_ (%) at the start of the study			
Mean ± SD	7.83 ± 1.95	6.51 ± 0.59	7.26 ± 1.50
Median	7.8	6.4	7.2
Min/max	5.9/9.8	5.8/7.4	5.8/8.8
*Lipid profile at the start of the study*			
Total cholesterol			
Mean ± SD	139 ± 29.70	160.4 ± 18.37	128.8 ± 27.30
Median	139	155.7	133.8
Min/max	118/160	139/184	88.30/164.3
High-density lipoprotein cholesterol			
Mean ± SD	38 ± 5.65	41.67 ± 13.22	43.14/15.57
Median	38	44.90	40.70
Min/max	31/42	25/58.20	24.30/67.40
Triglycerides			
Mean ± SD	146.5 ± 3.53	221.8 ± 165.3	162.4 ± 71.98
Median	146.5	157.3	148.1
Min/max	144/149	75.20/442	98.20/274.6
Basal Rutherford classification			
Stage 2, *n* (%)	–	1 (14)	–
Stage 3, *n* (%)	–	–	1 (16)
Stage 4, *n* (%)	2 (22)	–	2 (34)
Stage 5, *n* (%)	7 (78)	6 (86)	2 (34)
Stage 6, *n* (%)	–	–	1 (16)
Basal TcPO_2_ (mmHg)			
Mean ± SD	16.89 ± 9.88	8.28 ± 5.25	15 ± 9.35
Median	19	7	13
Min/max	4/30	4/18	6/28
Basal presence of ulcers (cm^***2***^***)***			
n (%)	8 (89)	6 (86)	5 (84)
Mean ± SD	1.643 ± 1.064	3.22 ± 3.63	7.95 ± 15.20
Median	1.735	2.57	1.5
Min/max	0.3/2.8	0.07/10.03	0.42/35.12
Basal visual pain scale			
Mean ± SD	8.66 ± 2.29	4.28 ± 3.49	8.25/0.95
Median	10	4	8.5
Min/max	3/10	0/9	7/9
Basal pain-free walking distance (meters)			
Mean ± SD	28.33 ± 40.77	445.7 ± 544.6	12.50 ± 25
Median	10	100	0
Min/max	0/100	20/1500	0/50
Basal quality of life			
Mean ± SD	8.70 ± 0.67	12.29 ± 2.43	10 ± 1.29
Median	9	12	10
Min/max	8/10	9/15	8/12

*Auto-BM-MNC* autologous bone marrow mononuclear cell, *allo-WJ-MSCs* allogenic Wharton jelly-derived mesenchymal stem cells, *SD* standard deviation and *TcPO*_*2*_ transcutaneous oxygen pressure

**Fig. 1:**
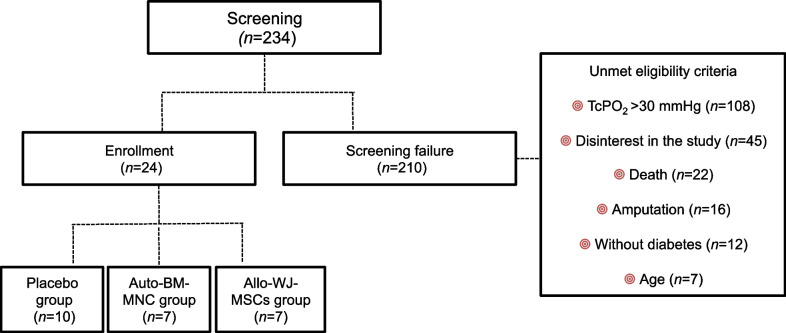
Participant flowchart. From a screening of 234 patients, 24 were enrolled as patients with CTLI. The study was composed of autologous bone marrow mononuclear cell group (auto-BM-MNC) (*n* = 7), allogenic Wharton jelly-derived mesenchymal stem cells group (allo-WJ-MSCs) (*n* = 7), and a placebo group (*n* = 10), as depicted on the left side. Reasons of screening failure were values of TcPO_2_ > 30 mmHg (*n* = 108), disinterest in the study (*n* = 45), death (*n* = 22), amputation (*n* = 16), non-diabetic participants (*n* = 12) and age (*n* = 7), as depicted on the right side

Two hundred thirty-four subjects with CLTI were screened, and 210 were excluded because of the unmet eligibility criteria such as TcPO_2_ > 30 mmHg values (*n* = 108), disinterest in the study (*n* = 45), death (*n* = 22), amputation (*n* = 16), non-diabetic condition (*n* = 12) and age (*n* = 7). The remaining twenty-four subjects (24 limbs) were randomly assigned as follows: placebo group, auto-BM-MNC, and allo-WJ-MSCs (Fig. 1).

### Treatments

In all participants, an eco-Doppler of the affected limb was performed, and immediate recognition of the three infrapatellar almost always occluded arteries with collateral vessel supply from the mid-leg to the distal leg segment (periareolar regions) such as anterior and posterior tibial arteries and peroneal artery. In addition, in some patients, we search for the dorsal pedal artery. Stem cell injections were made in a unique period by approaching a 27 g or 30 g needle connected to a 1 cc (insulin) syringe. Injections were made into the periadventitial arterial layer, 1–3 mm adjacent to the vessel wall. Fifteen stem cell injections were applied in the affected limb, in the periadventitial layer of these 3 or 4 previously mentioned arteries. All participants received local anesthetic (topic xylocaine gel).

Participants were randomly assigned to receive one of the following treatments:

(i) A placebo group (*n* = 10), which consisted of 15 injections of 1 mL of vehicle (1 mL saline solution with 2% of autologous serum) administered into the periadventitial layer of the arterial walls under eco-Doppler guidance (Additional file 1: Fig. S1) at day 0.

(ii) Auto-BM-MNC (*n* = 7) were obtained from diabetic patients. Surface markers of auto-BM-MNC were evaluated and the cells were positive for CD45^+^, CD34^+^, CD11b^+^, and HLA-DR^+^ and negative for CD73^−^, CD90^−^, and CD105^−^ (Supplementary material). Fifteen injections of 7.197 × 10^6^ ± 2.984 × 10^6^ cells/mL, each with 2% of autologous serum, were administered into the periadventitial layer of the arterial walls under eco-Doppler guidance at day 0.

(iii) Allo-WJ-MSCs (*n* = 7) were obtained from culturing the WJ from healthy cordon umbilical donors unrelated to the patient. Surface markers of WJ-MSCs were evaluated, and the cells were positive for CD73^+^, CD90^+^, and CD105^+^ and negative for CD45^−^, CD34^−^, CD11b^−^, and HLA-DR^−^ (Supplementary material). Fifteen injections of 1.333 × 10^6^ cells/mL, each with 5% human serum albumin serum, were administered into the periadventitial layer of the arterial walls under eco-Doppler guidance at day 0.

### Outcomes measures

#### Safety profile

The trial's primary endpoint was to determine the safety of auto-BM-MNC and allo-WJ-MSC administration; for that, we assessed the number of treatment-related adverse events (AEs) reported for each study group according to the Common Terminology Criteria for AE classification. AEs were defined as (i) local toxicity, including signs of local inflammation (swelling, warmth, impairment of function), worsening of ulcer, new ulcer, or hematomas after the cell administration, (ii) systemic toxicity as fever, allergies, and (iii) other AEs, graded according to the Common Terminology Criteria for AEs, expressed as maximum grade toxicity for tissue.

Secondary safety outcomes include any severe AEs post-treatment, defined as events leading to hospitalization, malignancy, amputation, persistent or significant disability, or death.

We documented AEs at each visit and described them in terms of incidence, severity, and relatedness with macroscopic changes in the leg.

#### Efficacy profile

The secondary endpoint of the trial was to evaluate the beneficial therapeutic effect of the treatments under study by: (i) Rutherford’s classification, (ii) the TcPO_2_, which was measured using a TCM4 monitor (Radiometer Medical ApS, Bronshoj, Denmark), (iii) the percentage of wound closure which was accurately measured using 3D laser technology (SilhouetteStar camera), (iv) the pain by assessing the visual analog scale [13], (v) the pain-free walking distance, (vi) revascularization (vii) limb-survival proportion during follow-up, and (viii) the life quality scale outcome (EQ-5D questionnaire). The follow-up visits were at 1, 3, 6, and 12 months.

Time elapsed to complete wound closure was determined when the wound bed became completely re-epithelialized and filled with new tissue.

The percentage of wound closure was calculated using the equation:

$$[({\text{original\,wound\,area}}-{\text{actual\,wound\,area}})/({\text{original\,wound\,area}})]\, \times 100.$$

### Preparation of investigational medicinal product (auto-BM-MNC and allo-WJ-MSCs)

Investigational Medicinal Products [14] were auto-BM-MNC, and allo-WJ-MSC suspensions, obtained from bone marrow aspirates from diabetic patients with CLTI and umbilical cord from healthy donors who were not HLA matched to the recipients, respectively.

Both auto-BM-MNC and allo-WJ-MSCs for this trial were processed and manufactured in a Good Manufacturing Practice (GMP) type Laboratory (Centro de Terapias Avanzadas FOSCAL, Colombia) under GMP conditions according to the Food and Drug Administration Guidance for Industry (current good tissue practice and additional requirements for manufacturers of human cells, tissues, and cellular and tissue-based products).

### Isolation and ex vivo culture of auto-BM-MNC

Bone marrow was obtained after informed consent from diabetic patients with CTLI and was aseptically stored in sterile Hank's Balanced Salt Solution (HBSS) (Gibco, Grand Island, NY, USA) supplemented with heparin (Fresenius Kabi, Chile). Briefly, mononuclear cells were separated by centrifugation in a Ficoll-Hypaque gradient (density 1.077 g/cm^3^, Sigma, St. Louis, MO) following the manufacturer's instructions. Next, the mononuclear cells were suspended in X-VIVO™ 10 Media (Lonza, Paisley, UK) supplemented with 2% heat-inactivated autologous serum and seeded at a concentration of 1 × 10^6^ cells/cm^2^. Cultures were maintained at 37 °C in a humidified atmosphere containing 5% carbon dioxide. After 24 h, cells were harvested and characterized for their administration.

### Isolation and ex vivo expansion of allo-WJ-MSCs

The umbilical cord was obtained after informed consent from healthy donors, and the acceptance of screening for pathogenic microorganisms (syphilis, hepatitis B virus, hepatitis C virus, human immunodeficiency virus type 1 and 2, human T-lymphotropic virus type 1 and 2, cytomegalovirus, and Toxoplasma Gondii).

The umbilical cord was aseptically stored in sterile HBS. Allo-WJ-MSCs were isolated by enzymatic method. In brief, blood, vessels, and arteries were removed from the umbilical cord. Then, the remaining tissue was cut into smaller pieces which were placed in a collagenase enzymatic solution at 1 mg/mL for four hours, followed by incubation in trypsin-ethylene diamine-tetra-acetic-acid (EDTA) (Gibco, Paisley, UK) solution at 2.5 mg/mL, during 15 min in a humidified environment 37 °C containing 5% carbon dioxide. After incubation, the digested suspension was collected by gravity in a 50 mL conical tube, diluted, and centrifuged at 2000 revolutions per minute for 40 min at 18 ºC. The mononuclear cells were suspended in Minimum Essential Medium Eagle (MEM) (Gibco, Paisley, UK) supplemented with 10% human derivatives from platelet-rich plasma (hD-PRP) obtained from donors with type AB blood, 1% gentamicin, and 2 mM L-glutamine (Gibco, Grand Island, NY, U.S.), and seeded at a concentration of 0.5 × 10^6^ cells/cm^2^. After 72 h, non-adherent cells were removed, and fresh medium was added to the cells. Cultures were maintained at 37 °C in a humidified atmosphere containing 5% carbon dioxide. One week later, when the monolayer of adherent cells reached confluence, cells were trypsinized (0.25% trypsin and 2.65 mM EDTA), washed, resuspended in supplemented MEM, and subcultured at a concentration of 7,000 cells/cm^2^. When reached 70–80% confluence, cells were detached by treatment with (0.25% trypsin and 2.65 mM EDTA).

Finally, the cells were expanded in vitro to manufacture the required number of cells for the treatment. In the process, a donor master cell bank and working cell bank were maintained as a source of MSCs for future manufacturing purposes.

The working cell bank was upscaled further to produce the IMP at passage 3, which was used for the pilot study. Once the desired number of cells was produced, aliquots of samples were provided for quality control testing. These include complete characterization according to the International Society for Cellular Therapy Guidelines [15] by flow cytometry and differentiation capacity.

At passage 3, allo-WJ-MSCs were harvested and tested to confirm that were devoid of any microbial contaminants (mycoplasma, endotoxin (≤ 0.5 EU/mL), and aerobic anaerobic and fungi culture).

### Preparation and immunophenotypic analysis of allo-WJ-MSCs

At passage 3, when cell cultures reached 80% confluence, adherent cells were detached by treatment with (0.25% trypsin and 2.65 mM EDTA). Live cells were counted using trypan blue staining and a hemocytometer. The release criteria for clinical use of allo-WJ-MSCs comprised the absence of macroscopic clumps, contaminating pathogenic microorganisms, and viability > 95%, with an identity and purity pattern characterized by ≥ 95% positivity for CD73 clone AD2 (PerCP-Cy5.5-conjugated, BD Biosciences TM), CD90 clone 5E10 (FITC-conjugated, eBioscience TM), and CD105 clone MJ7/18 (eFluor450-conjugated, eBioscience TM), and negativity (≤ 2%) for the expression of CD45 clone HI30 (PE-Cyanine 7 conjugated, eBioscience TM), CD34 clone 4H11 (PE-conjugated, eBioscience TM), CD11b clone ICRF44 (APC-conjugated, eBioscience TM), CD14, and Human Leukocyte Antigen-DR isotype (HLA-DR) clone G46-6 (APC-H7-conjugated, BD Biosciences TM). Next, the cell pellet was rinsed once with a staining buffer and resuspended in a loading buffer, and a total of 10,000 events were analyzed per condition. Flow cytometry analysis was performed using an Amnis® CellStream® benchtop system (Luminex, Millipore).

The genomic stability of allo-WJ-MSCs was evaluated by karyotyping analysis (data not shown).

### Statistical analysis

Data were reported as mean ± standard deviation. Once statistical normality was checked, a comparison of experimental groups was performed using a Kruskal–Wallis one-way analysis followed by Dunn’s Multiple Comparison Test to test significant differences (*p* < 0.05) in quantitative variables among treatment groups (placebo, auto-BM-MNC or allo-WJ-MSCs) at baseline and during follow-up. The association or independence of categorical variables was compared using Pearson's chi-square test, and a *p* < 0.05 value was accepted as statistically significant. Limb-survival proportion analysis was calculated according to the Kaplan–Meier function. A *p* value < 0.05 was considered statistically significant. Stat Graph Prism 5.0 and STATA 15 software were used for statistical analysis.

## Results

### Participant demographic characteristics

We screened 234 individuals with CLTI, from which 24 were eligible and randomly assigned to receive one of the treatments under study (Fig. 1). Mostly, the participants enrolled in each group had similar baseline characteristics of age, hemoglobin A1c, and lipid profile; in fact, the groups had no statistically significant differences among them (Table 2).

### Safety profile

The cell administration procedure was well tolerated, with no or mild discomfort. The cell product injection into the periadventitial arterial walls generated no complications during the first 72 h. Furthermore, no participant exhibited any or serious AEs related to the therapy with auto-BM-MNC or allo-WJ-MSCs (such as infection at the injection site, immunological rejection, or tumor generation) during follow-up.

In addition, after bone marrow aspiration, we observed no bleeding, infection, or other complications. Moreover, the separation of the aspirate by Ficoll, its subsequent cell culture, as well as isolation and ex vivo expansion of allo-WJ-MSCs went smoothly.

Nevertheless, four deaths, non-related to the treatment, arose while the patients still were participating in the trial. Two participants died in the auto-BMNC group, one from complications resulting from an acute myocardial infarction (follow-up at ten months) and another due to COVID-19 (follow-up at five months). In the allo-WJ-MSC group, one participant died due to pleural effusion (follow-up at ten months), and the other died due to cardiac disorder (follow-up at ten months). Similarly, in the placebo group, one of the participants died due to CLTI complications (follow-up at three months).

### Efficacy profile: clinical outcomes

#### Rutherford’s classification

**Fig. 2:**
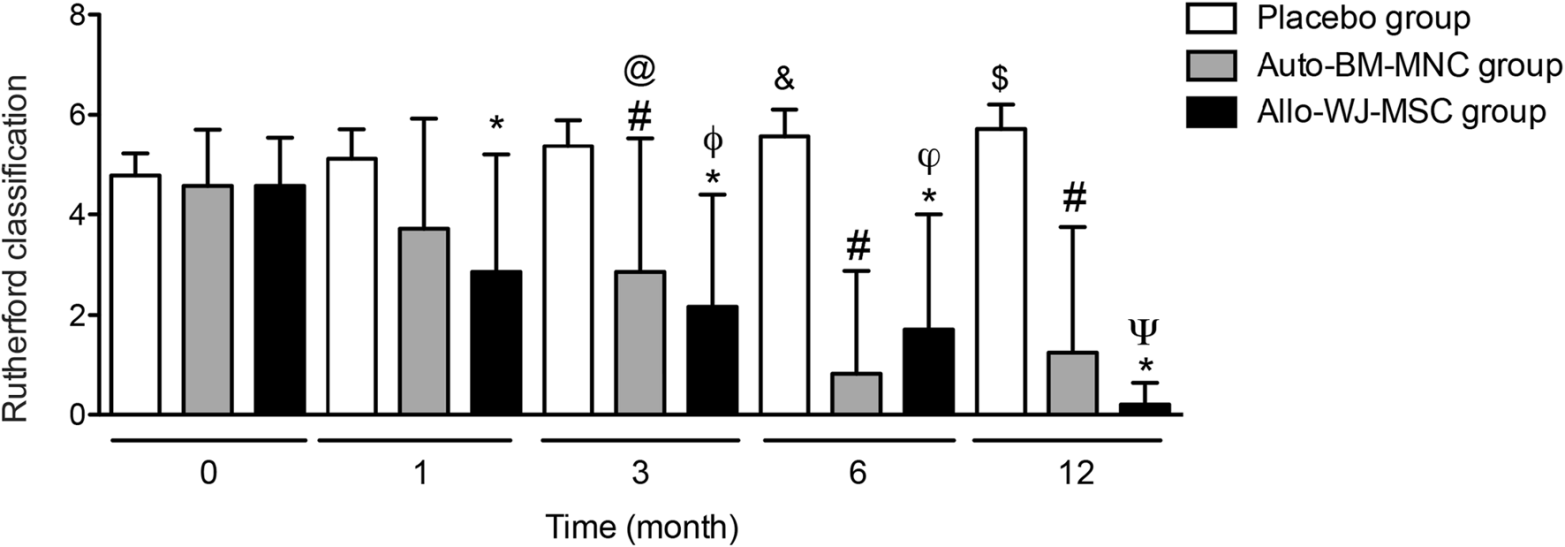
Severity Rutherford’s classification decreased in auto-BM-MNC and allo-WJ-MSC—treated diabetic patients with CTLI. Rutherford’s classification decreased in the auto-BM-MNC and allo-WJ-MSC groups starting from the first month (Rutherford < 3), which persisted until 12 months after cell-based therapy, unlike the placebo group, whose participants increased their classification to a more severe stage of the disease (Rutherford 6). Measurements are expressed as mean ± standard deviation. Significant differences between: placebo group versus auto-BM-MNC group (#) (*p* < 0.05); placebo group versus allo-WJ-MSC group (*) (*p* < 0.05); auto-BM-MNC group pre-treatment versus auto-BM-MNC group three months post-treatment (@) (*p* < 0.05); allo-WJ-MSC group pre-treatment versus allo-WJ-MSC group three months post-treatment (*ϕ*) (*p* < 0.05); placebo group pre-treatment versus placebo group six months post-treatment (&) (*p* < 0.05); allo-WJ-MSC group pre-treatment versus allo-WJ-MSC group six months post-treatment (*φ*) (*p* < 0.05); placebo group pre-treatment versus placebo group twelve months post-treatment ($) (*p* < 0.05) and allo-WJ-MSC group pre-treatment versus allo-WJ-MSC group twelve months post-treatment (Ψ) (*p* < 0.05). Auto-BM-MNC, autologous bone marrow mononuclear cell and allo-WJ-MSCs, allogenic Wharton jelly-derived mesenchymal stem cells

We used Rutherford’s classification as a numerical objective evaluation to determine the CLTI severity. We found a statistically significant decrease in Rutherford’s classification in the auto-BM-MNC and allo-WJ-MSC groups from the first month (Rutherford < 3), which persisted until 12 months after administrating the cell-based therapy, unlike the placebo group, whose participants increased their classification to a more advanced stage of the disease (Rutherford 6) (Fig. 2).

#### TcPO_2_

**Fig. 3:**
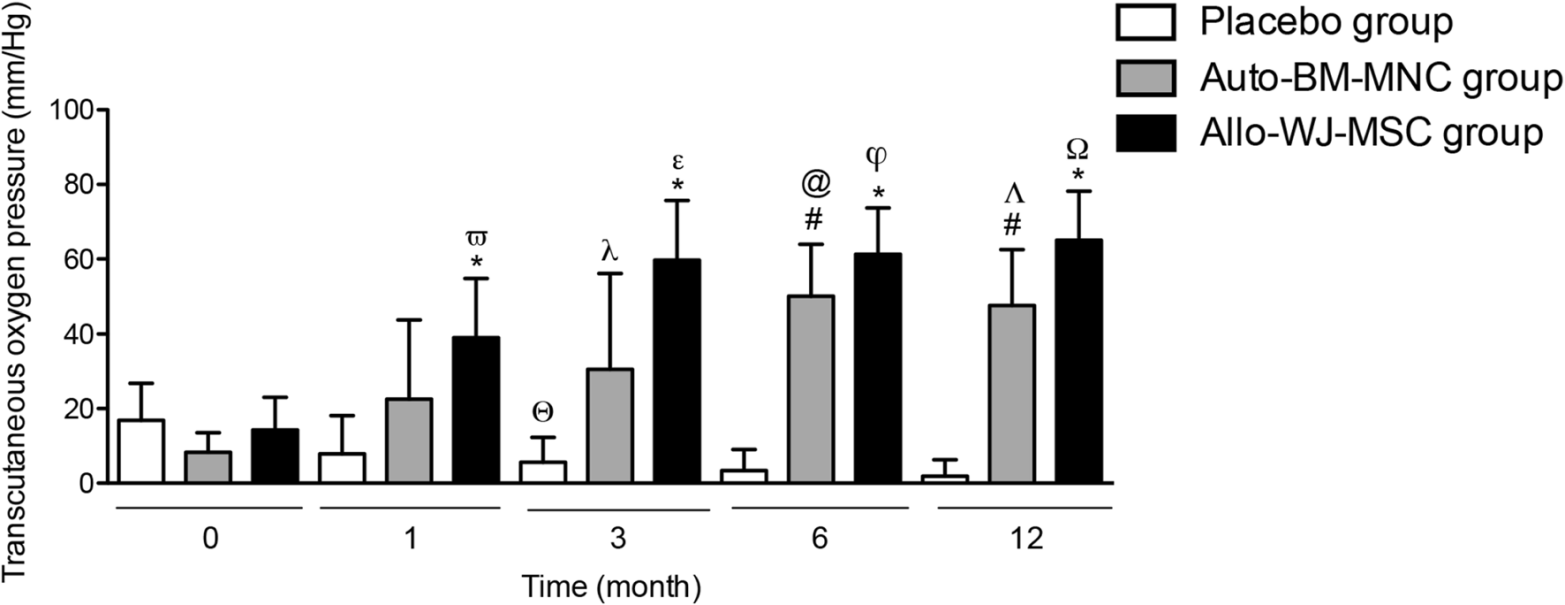
Transcutaneous oxygen saturation of the affected limb improved in auto-BM-MNC and allo-WJ-MSC-treated diabetic patients with CTLI. TcPO_2_ improved in the auto-BM-MNC and allo-WJ-MSC groups from the first month, which persisted until 12 months after cell-based therapy, unlike the placebo group, whose participants decreased their TcPO_2_. Measurements are expressed as mean ± standard deviation. Significant differences between: placebo group versus auto-BM-MNC group (#) (*p* < 0.05); placebo group versus allo-WJ-MSC group (*) (*p* < 0.05); allo-WJ-MSC group pre-treatment versus allo-WJ-MSC group one month post-treatment (*ϖ*) (*p* < 0.05); placebo group pre-treatment versus placebo group three months post-treatment (*θ*) (*p* < 0.05); auto-BM-MNC group pre-treatment versus auto-BM-MNC group three months post-treatment (*λ*) (*p* < 0.05); allo-WJ-MSC group pre-treatment versus allo-WJ-MSC group three months post-treatment (*ε*) (*p* < 0.05); auto-BM-MNC group pre-treatment versus auto-BM-MNC group six months post-treatment (@) (*p* < 0.05); allo-WJ-MSC group pre-treatment versus allo-WJ-MSC group six months post-treatment (*φ*) (*p* < 0.05); auto-BM-MNC group pre-treatment versus auto-BM-MNC group twelve months post-treatment (Λ) (*p* < 0.05) and allo-WJ-MSC group pre-treatment versus allo-WJ-MSC group twelve months post-treatment (Ω) (*p* < 0.05). Auto-BM-MNC, autologous bone marrow mononuclear cell and allo-WJ-MSCs, allogenic Wharton jelly-derived mesenchymal stem cells

In order to assess the transcutaneous oxygen saturation, we measured it on the affected limb’s foot at the beginning of the study and at month 1, 3, 6, and 12 of follow-up. First, at baseline, we observed a TcPO_2_ of 8.28 mmHg ± 5.25 in the auto-BM-MNC group, 14.29 mmHg ± 8.75 in the allo-WJ-MSC group, and 16.89 mmHg ± 9.88 in the placebo group. One month after transplantation, the TcPO_2_ levels of the limbs treated with auto-BM-MNC and allo-WJ-MSC increased significantly (mean: 22.57 mmHg ± 21.20 vs. 39 mmHg ± 15.76, respectively) compared to those in the placebo group (7.85 mmHg ± 10.29). Three months after cell infusion, the TcPO_2_ reached values of 30.43 mmHg ± 25.65 and 59.67 mmHg ± 16.05 in the auto-BM-MNC and allo-WJ-MSC groups, respectively; while remained low in the placebo group (5.66 mmHg ± 6.65). At month six, the mean TcPO_2_ was 50 ± 13.98 versus 61.29 ± 12.43 mmHg in the auto-BM-MNC and allo-WJ-MSC, respectively, and 3.40 ± 5.63 mmHg in the placebo group. This growth persisted after 12 months of transplantation in auto-BM-MNC and allo-WJ-MSC (47.50 ± 15.02 vs. 65 ± 13.21 mmHg) groups, unlike to placebo group (1.88 ± 4.37 mmHg) (Fig. 3).

#### Wound closure

**Fig. 4:**
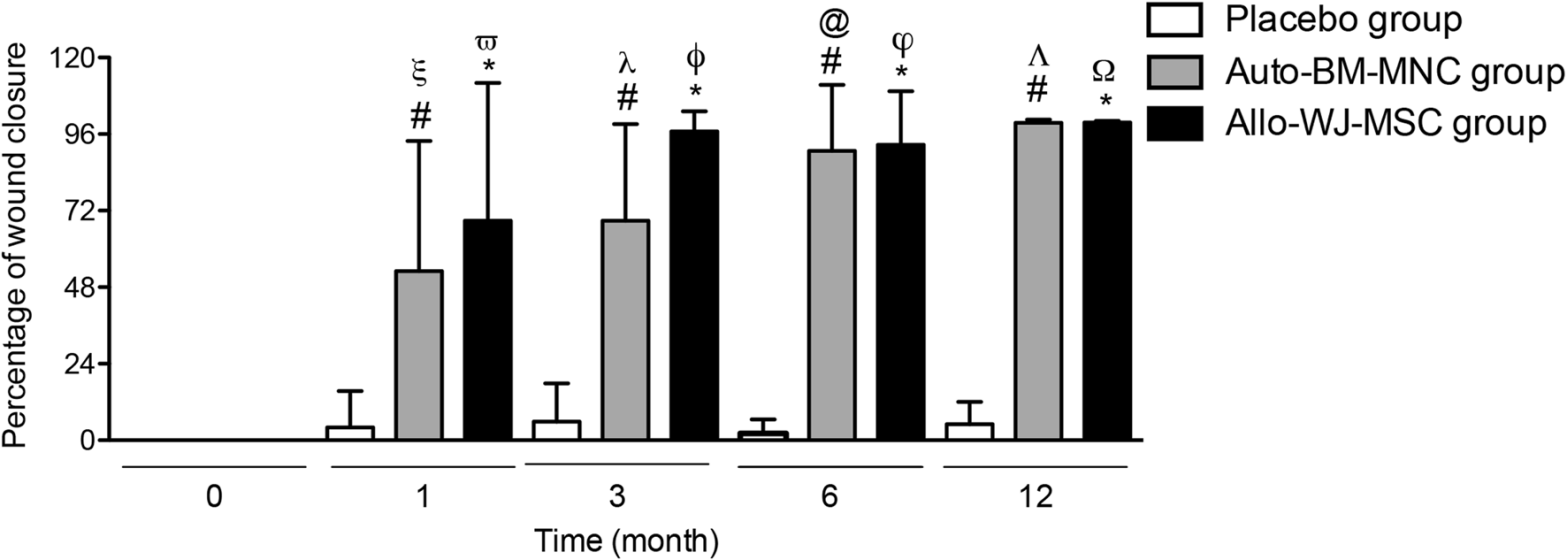
Diabetic patients with CTLI treated with auto-BM-MNC, or allo-WJ-MSCs, exhibited satisfactorily wound closure of the affected limb. The percentage of wound closure in participants treated with auto-BM-MNC or allo-WJ-MSCs was higher than in those treated with the placebo solution starting from the first month of the cell-based therapy, compared to the placebo group. Measurements are expressed as mean ± standard deviation. Significant differences between: placebo group versus auto-BM-MNC group (#) (*p* < 0.05); placebo group versus allo-WJ-MSC group (*) (*p* < 0.05); auto-BM-MNC group pre-treatment versus auto-BM-MNC group one month post-treatment (*ξ*) (*p* < 0.05); allo-WJ-MSC group pre-treatment versus allo-WJ-MSC group one month post-treatment (*ϖ*) (*p* < 0.05); auto-BM-MNC group pre-treatment versus auto-BM-MNC group three months post-treatment (*λ*) (*p* < 0.05); allo-WJ-MSC group pre-treatment versus allo-WJ-MSC group three months post-treatment (*ϕ*) (*p* < 0.05); auto-BM-MNC group pre-treatment versus auto-BM-MNC group six months post-treatment (@) (*p* < 0.05); allo-WJ-MSC group pre-treatment versus allo-WJ-MSC group six months post-treatment (*φ*) (*p* < 0.05); auto-BM-MNC group pre-treatment versus auto-BM-MNC group twelve months post-treatment (Λ) (*p* < 0.05) and allo-WJ-MSC group pre-treatment versus allo-WJ-MSC group twelve months post-treatment (Ω) (*p* < 0.05). Auto-BM-MNC, autologous bone marrow mononuclear cell and allo-WJ-MSCs, allogenic Wharton jelly-derived mesenchymal stem cells

Wound closure started to satisfactorily appear after one month of treatment with either auto-BM-MNC or allo-WJ-MSCs, compared to the placebo group. The percentage of wound closure in participants treated with auto-BM-MNC or allo-WJ-MSC was higher than those treated with the placebo solution (Fig. 4). Specifically, after one month of treatment, the auto-BM-MNC and the allo-WJ-MSC groups achieved a statistically significant reduction of wound closure compared to time 0 (53.02 ± 40.85% and 68.86 ± 43.23%, respectively); in contrast, the placebo group only exhibited a 4.03 ± 11.42% of reduction in wound surface area. At month three, we found statistically significant differences between the auto-BM-MNC and the allo-WJ-MSC versus the placebo group (68.35 ± 30.33% vs. 96.89 ± 6.22% vs. 5.83 ± 12.01%, respectively). At month six, we similarly observed significant differences between the auto-BM-MNC and the allo-WJ-MSC versus the placebo group (90.74 ± 20.70% vs. 92.68 ± 16.76% vs. 2 ± 4.47%, respectively).

Furthermore, the data showed that patients with CLTI treated with auto-BM-MNC or allo-WJ-MSC reached more than 50% of wound closure after one month; in contrast, participants of the placebo group never achieved wound closure (Fig. 4).

At the end of the follow-up, the ulcer healing ratio was 7 of 7, 100%, for the auto-BM-MNC and allo-WJ-MSC groups. This value was significantly higher than the ratio of the placebo group (0 of 10, 0%).

#### Pain evaluation

**Fig. 5:**
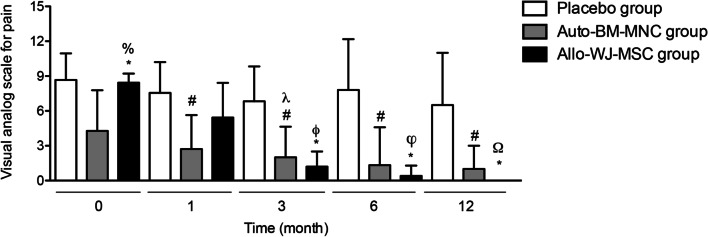
Pain intensity at rest decreased dramatically in the auto-BM-MNC and allo-WJ-MSC-treated diabetic patients with CTLI. Pain decreased in participants treated with auto-BM-MNC or allo-WJ-MSC approaches from the first month compared to the placebo group. Measurements are expressed as mean ± standard deviation. Significant differences between: placebo group versus auto-BM-MNC group (#) (*p* < 0.05); placebo group versus allo-WJ-MSC group (*) (*p* < 0.05); auto-BM-MNC group versus allo-WJ-MSC group (%) (*p* < 0.05); auto-BM-MNC group pre-treatment versus auto-BM-MNC group three months post-treatment (*λ*) (*p* < 0.05); allo-WJ-MSC group pre-treatment versus allo-WJ-MSC group three months post-treatment (*ϕ*) (*p* < 0.05); allo-WJ-MSC group pre-treatment versus allo-WJ-MSC group six months post-treatment (*φ*) (*p* < 0.05); and allo-WJ-MSC group pre-treatment versus allo-WJ-MSC group twelve months post-treatment (Ω) (*p* < 0.05). Auto-BM-MNC, autologous bone marrow mononuclear cell and allo-WJ-MSCs, allogenic Wharton jelly-derived mesenchymal stem cells

We measured the pain intensity at rest by Visual Analogue Scale (VAS) at the beginning of the study and after 1, 3, 6, and 12 months of follow-up. The pain evaluation measures showed statistically significant differences between the auto-BM-MNC or allo-WJ-MSC approaches compared to the placebo group at all the times of follow-up (Fig. 5). In detail, we found a mean baseline pain VAS in the auto-BM-MNC, allo-WJ-MSC, and placebo groups of 4.28 ± 3.49 versus 8.42 ± 0.78 versus 8.66 ± 2.29, respectively, which improved at month one to 2.71 ± 2.92 and 5.42 ± 2.99 in the auto-BM-MNC and allo-WJ-MSC groups, respectively, unlike placebo group in which remained high (7.55 ± 2.65). At month three, the mean pain VAS in the auto-BM-MNC and allo-WJ-MSC groups were 2 ± 2.64 versus 1.20 ± 1.30, respectively, while the placebo group continued being elevated *(*6.83 ± 2.99). At month six, the pain intensity at rest continued improving in the auto-BM-MNC and allo-WJ-MSC groups (1.33 ± 3.26 vs. 0.40 ± 0.89, respectively) versus the placebo group (7.80 ± 4.38). Finally, at month 12, the pain intensity decreased even more in the cell-treated groups in the autologous group (1 ± 2) and disappeared in the allogenic group (0 ± 0)*;* however, it remained elevated in the placebo group (6.50 ± 4.50).

#### Pain-free walking distance (meters)

We observed an increase in the pain-free walking distance (PWD) from 445.70 ± 544.60 m to 800 ± 675.80 m in the auto-BM-MNC group, and from 37.14 ± 46.45 m to 400 ± 420.3 m in the allo-WJ-MSC group at month one after the cell administration. In contrast, PWD decreased from 28.33 ± 40.77 m to 15.56 ± 32.45 m in the placebo group. After three months of having administered the cell-based therapy, the PWD continued increasing in the auto-BM-MNC and allo-WJ-MSC groups (928.60 ± 769.70 m vs. 635.70 ± 539.10 m), compared to the placebo group, in which the distance decreased (15 ± 34.64 m).

At month six, the PWD was 725 ± 644.90 m and 654.30 ± 530.20 m in auto-BM-MNC and allo-WJ-MSC groups, respectively; while was 13.75 ± 35.03 m in the placebo group.

**Fig. 6:**
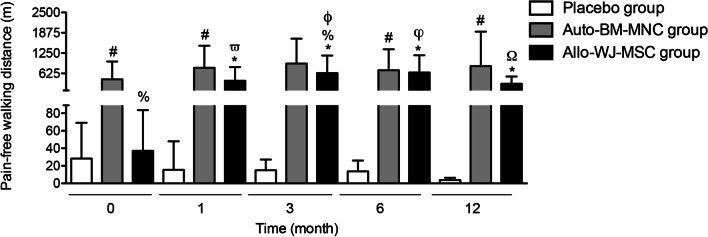
Diabetic patients with CTLI showed drastically reduced pain-free walking after auto-BM-MNC or allo-WJ-MSC treatment. Pain-free walking distance diminished from the first month of treatment with auto-BM-MNC and allo-WJ-MSCs, unlike in the placebo group. Measurements are expressed as mean ± standard deviation. Significant differences between: placebo group versus auto-BM-MNC group (#) (*p* < 0.05); placebo group versus allo-WJ-MSC group (*) (*p* < 0.05); auto-BM-MNC group versus allo-WJ-MSC group (%) (*p* < 0.05); allo-WJ-MSC group pre-treatment versus allo-WJ-MSC group one month post-treatment (*ϖ*) (*p* < 0.05); allo-WJ-MSC group pre-treatment versus allo-WJ-MSC group three months post-treatment (*ϕ*) (*p* < 0.05); allo-WJ-MSC group pre-treatment versus allo-WJ-MSC group six months post-treatment (*φ*) (*p* < 0.05) and allo-WJ-MSC group pre-treatment versus allo-WJ-MSC group twelve months post-treatment (Ω) (*p* < 0.05). Auto-BM-MNC, autologous bone marrow mononuclear cell and allo-WJ-MSCs, allogenic Wharton jelly-derived mesenchymal stem cells

At the end of the follow-up, the PWD between the auto-BM-MNC and the allo-WJ-MSC groups was significantly higher than the placebo group (850 ± 1061 m vs. 306 ± 225 m vs. 3.75 ± 7.44 m, respectively) (Fig. 6).

#### Revascularization and amputation-free survival

No limb revascularization was necessary for the auto-BM-MNC (0 of 7) and allo-WJ-MSC (0 of 7) groups, while two participants of the placebo group had to receive revascularization (2 of 10).

**Fig. 7:**
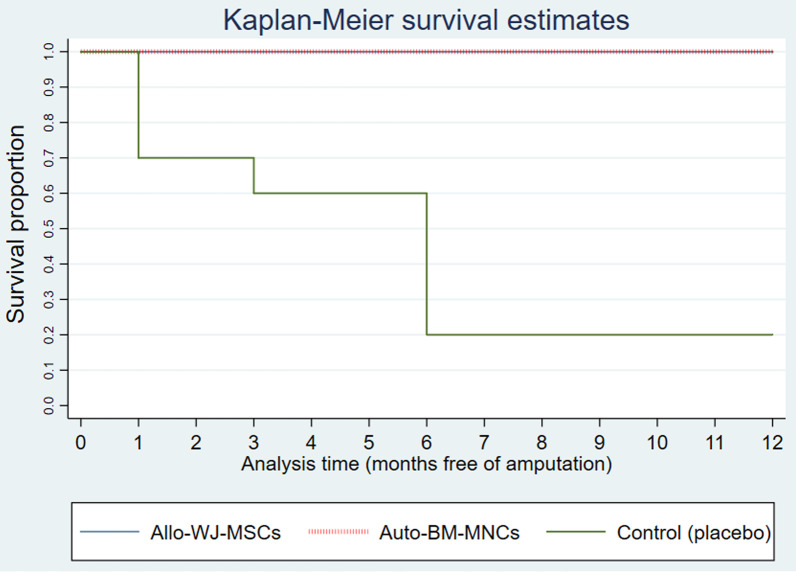
Diabetic patients with CTLI conserved their limbs after the treatment with auto-BM-MNC or allo-WJ-MSCs. Amputation-free survival analysis showed that patients with CLTI treated with auto-BM-MNC and allo-WJ-MSCs conserved 100% of their limbs during 12 months of the follow-up compared to the placebo group, where 60% of participants underwent limb amputation at different times. Auto-BM-MNC, autologous bone marrow mononuclear cell and allo-WJ-MSCs, allogenic Wharton jelly-derived mesenchymal stem cells

The amputation-free survival analysis between auto-BM-MNC and allo-WJ-MSC versus placebo groups showed that patients with CLTI treated with auto-BM-MNC and allo-WJ-MSCs conserved 100% of their limb after 12 months of follow-up compared to the placebo group, where 60% of participants underwent limb amputation at different times (Fig. 7).

Amputation-free survival analysis was adjusted by age and baseline hemoglobin A1c; however, these variables did not interfere with the outcome.

#### Quality of life (EQ-5D questionnaire)

**Fig. 8:**
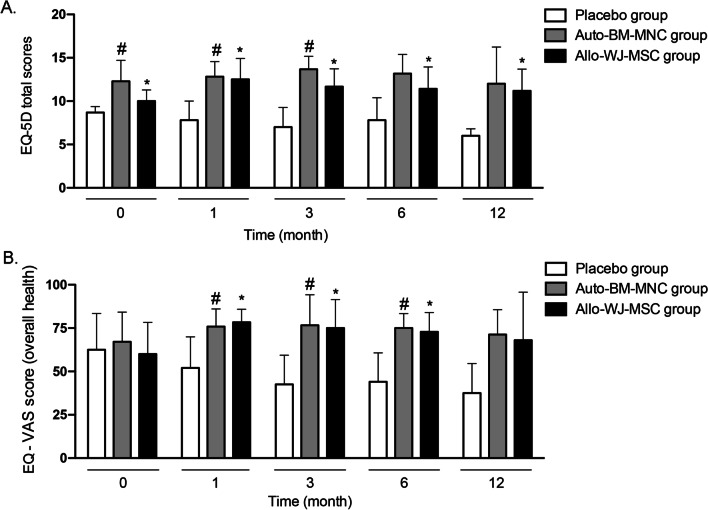
Diabetic patients with CTLI improved their quality of life after the treatment with auto-BM-MNC or allo-WJ-MSCs. **A** EQ-5D profile of the participants showed statistically significant differences among auto-BM-MNC, allo-WJ-MSC, and placebo groups, before and after treatment. **B** The self-assessed measure of overall health (EQ-VAS) showed improvements in patients’ health problems from treatment. Measurements are expressed as mean ± standard deviation. Significant differences between: placebo group versus auto-BM-MNC group (#) (*p* < 0.05); placebo group versus allo-WJ-MSC group (*) (*p* < 0.05). Auto-BM-MNC, autologous bone marrow mononuclear cell and allo-WJ-MSCs, allogenic Wharton jelly-derived mesenchymal stem cells

Patients with CLTI treated with auto-BM-MNC or allo-WJ-MSCs presented an enhancement of their life quality, as shown by the data of the EQ-5D profile (Fig. 7). There were statistically significant differences between auto-BM-MNC, allo-WJ-MSC, and placebo groups, before and after the treatments. Furthermore, even after one month post-treatment, the participant’s quality of life improved in the auto-BM-MNC and allo-WJ-MSC groups, while it deteriorated in the placebo group (Fig. 8A).

The improvement in EQ-5D was primarily due to the following dimensions: mobility, physical functioning, usual activities, self-care, pain, and no anxiety or depression.

All participants reported satisfaction with the results of cell-based therapy and recognized that the pain relief of their treated leg enabled them to enhance their usual activity level, including walking and presenting a significant reduction in the use of analgesics. Additionally, the EQ-5D score revealed stabilization or improvement of symptoms compared to the placebo group during the follow-up. Furthermore, the self-assessed measure of overall health (visual analog scale (EQ-VAS)) showed improvements in patients’ health problems from treatment (Fig. 8B).

## Discussion

Patients afflicted with CLTI have a poor quality of life and a high rate of limb loss [16]. Until now, revascularization of the ischemic limb, either by endovascular or open surgical approaches, has been the mainstay of therapy. However, 25% to 40% of people with CLTI are not suitable for it or have failed previous revascularization therapy, and the mortality rate remains high [17–19]; therefore, less invasive medical therapies that be effective in the treatment of CLTI are desirable.

In this context, new strategies such as regenerative medicine have enabled the development of therapeutic angiogenesis through recombinant proteins, gene transfer, or stem cells [5]. Nevertheless, the trials that only use recombinant proteins (growth factors) cannot provide the essential factors that patients with CLTI require. In the case of gene therapy, there may be relevant risks such as an increasing neo-vascularization in undesired tissue, malignant cell transformation and inflammation. Additionally, increased angiogenesis can destabilize atherosclerotic plaques, leading to arterial thrombosis. In particular, phase II and III clinical trials on angiogenic gene therapy showed mixed outcomes of positive and negative final results; thus, the role of gene therapy in vascular occlusive disease prevails unresolved [5, 20]. According to this, when comparing the approaches based on proteins or genes *vs* cell-based therapies, the last ones are more beneficial because of their natural vasculogenic features and their paracrine impact [1].

Initial preclinical and small pilot clinical studies have demonstrated promising effects of cell therapy in PAD and CLTI. Particularly, the results of these studies suggest that most stem cell therapies can increase blood flow at the transplantation site by promoting angiogenesis and neo-vascularization through a direct effect of the administered cells on the vasculature, or by secretion of pro-angiogenic factors and modulation of the local immune response, which prevents amputation in patients with CLTI [12]. However, it is unclear whether BM-MNC or MSCs are more effective in both PAD and CLTI. In fact, some clinical trials gave inconsistent results revealing that BM-MNC are ineffective and MSCs may be superior [12, 18]. Based on this, the present work compared the effect of auto-BM-MNC versus allo-WJ-MSCs in patients with CLTI. To our knowledge, this is the first study conducted in Colombia that compared the security and therapeutic potential of both cell-based therapies in diabetic patients with CLTI.

A crucial concern with stem cell therapy is its safety profile. Nevertheless, most of the published preclinical and clinical trials have reported that stem cells are safe to treat numerous injuries and diseases, including lower extremity vascular disease [21, 22].

The present study did not detect serious AEs, like malignancy, infection, organ system complications, or acute toxicity related to auto-BM-MNC or allo-WJ-MSC injections used to treat CLTI. In contrast, the participants receiving the placebo solution presented less ulcer healing and higher amputation rates. These findings were consistent with the evidence from numerous clinical trials that evaluated the safety of BM-MNC or MSC-based therapy in CLTI [2, 23, 24].

Regarding the effectiveness profile of the cell-based therapy, few clinical studies have aimed to compare the efficacy of different stem cells in treating CLTI in diabetic patients.

Several studies have reported that diabetic patients showed both a marked depletion of CD34^+^ and a functional impairment of cultured EPCs [9–11]. These findings suggest that the reduced number and dysfunction of EPCs in diabetes mirror an insufficient endogenous regenerative capacity, which contributes to the development of vascular complications.

Although our results strongly indicate that auto-BM-MNC and allo-WJ-MSCs increase limb’s blood flow and improve claudication symptoms of limb ischemia, we observe more promising results with allo-WJ-MSCs than auto-BM-MNC. Remarkably, in the clinical parameters that were evaluated, we observed an improvement in Rutherford’s classification, a significant increase in TcPO_2_ (values > 30 mm Hg), enhanced chronic ischemic ulcer healing, relief from a PWD, and increased amputation-free survival rates, which correlated with a recovery of the blood supply. In contrast, in the placebo group, the participants displayed more significant/higher amputation and surgical revascularization rates.

On the other hand, a number of preclinical and clinical studies have not demonstrated the clear benefits of auto-BM-MNC in various cardiovascular diseases [25]. Direct effects of cell aging on tissue repair capabilities are one of the strongest predictors of a lack of clinical response to auto-BM-MNC therapy; for instance, aged ECs are not only less effective at migration, but also more prone to become senescent, and have an altered secretion profile that contributes to the development of vascular complications [25].

Furthermore, the expected therapeutic angiogenesis using autologous BM-derived stem cells displays several disadvantages. Among them are the considerably limited amount of bone marrow obtained, and the procedure is painful for the patients. Besides, it may require general anesthesia, which can be life-threatening for patients with CLTI, who are already at elevated risk for difficulties due to their advanced age and cardiovascular disease. Similarly, the migration of circulating BM-derived stem cells is inefficient and significantly lower in patients with CLTI than in healthy subjects due to extended pro-inflammatory stimuli [20].

Recently, different studies have shown that autologous BM-MSC transplantation in patients with CLTI may have a risk of presenting karyotypic aberrations. Nonetheless, it is still not entirely understood if these abnormalities are innate of patients’ cells or have been originated during cell culture [26]. In this context, several studies have demonstrated that autologous MSCs obtained from patients suffering from inflammatory or degenerative diseases have variability in their biological and functional properties, provoking deleterious consequences for the host when dealing with host signals [27, 28]. Mainly, MSCs derived from individuals with atherosclerosis develop a pro-inflammatory secretome by the production of inflammatory cytokines such as IL6, IL8, and MCP1, reversing their naturally immunosuppressive properties [29]. Thus, the allogeneic cells allow for the best approach in cell-based therapy.

In this regard, studies that used Buerger's disease and ischemic limb disease animal models, demonstrated that umbilical cord blood-derived MSCs (UCB-MSCs) regenerated arterioles and promoted the differentiation into, UCB-MSCs offer various advantages due to (i) the newborn cell immaturity compared to adult stem cells and (ii) the ability to prevent immune reactions. In addition, the UCB-MSCs are less vulnerable to the attack of the recipient’s body than BM-derived stem cells. Several clinical trials have demonstrated that intramuscular administration of UCB-MSCs conduces to arterial reconstruction or prevention of arterial obstruction, decreases pain at rest and speeds up the healing process of ischemic ulcers. These studies proposed that growth factors or pain releasers secreted by implanted stem cells before vessel generation in ischemic regions, may be responsible for pain relief [30, 31].

Another clinical trial that used UCB-MSCs demonstrated the creation of new collateral arteries by computerized tomography angiography; this change was more apparent in the microvascular network than in the macrovascular network. Furthermore, compared to pre-treatment levels, the percentages of CD3^+^ CD8^+^ lymphocytes were significantly raised following treatment with UCB-MSCs, while percentages of CD3^+^ CD4^+^ lymphocytes and CD3-CD16/CD56^+^ NK cells were significantly reduced [20].

The MSC’s therapeutic role in patients with CLTI has been attributed to their unique biological features. MSCs can promote angiogenesis, reduce fibrosis, restore collagen balance, decrease immune cell activities, and undergo EC differentiation [32]. Likewise, MSCs and ECs engage in close cross-talk; specifically, MSCs stimulate the growth and relocation of ECs to initiate the early phases of angiogenesis, and lessen the permeability of the EC monolayer; this effect can be by both direct cell–cell contact and release of paracrine factors [33].

Different studies have shown that the MSC involvement in maintaining structures of neovessels in vivo is through several molecular pathways. Among those, the Wnt pathways are essential in adjusting MSC differentiation, proliferation, and migration. WNT4 activation in MSC increases blood flow. In particular, frizzled-related protein-1, a Wnt modulator secreted by MSCs, promotes angiogenesis by increasing MSC integration into neovessels, which implies that specific molecular targets are responsible for MSC engraftment into the vasculature, while TGF-β signaling regulates MSC differentiation into pericytes [34–36].

Regarding the best form of cell administration, most therapeutic trials addressing CLTI have focused on intramuscular cell administration as a more feasible and less harmful approach, demonstrating safety and effectiveness [33, 37]. However, in our study, auto-BM-MNC and allo-WJ-MSCs were administered into the periadventitial layer of the arterial walls; which generates a cell repository in situ.

Other administration routes are intravascular, which is more invasive and harmful since involves administering contrast materials that are injected into a vein, but is not recommended in patients with chronic renal disease [38]. Systemic delivery, such as intravenous (IV) or intra-arterial (IA) infusion, is used less frequently [39]. Notably, the IV route has shown many entrapments and lung embolus development [40]. Furthermore, the IV route inhibits EC proliferation and angiogenesis via cell–cell contact through modulation of the cadherin/catenin signaling pathways [41]. Other studies have reported thrombogenic events during IV MSC infusion [42, 43].

IA injection entails the danger of injury to the nerves and arteries, vessel wall dissection, and dislodgment of atherosclerotic plaques [37]. In our case, the periadventitial injection was easy to use, less invasive and demonstrated safety and effectiveness.

Another point of concern is the cell dosage to apply, which can vary according to the cell type/source [1]. Although the ideal number of cells to employ for angiogenesis is unknown, and there are limited studies on cell dose, in our study were administrated 15 injections of either BM-MNC (7.197 × 10^6^ ± 2.984 × 10^6^ cells/mL each injection) or allo-WJ-MSCs (1.333 × 10^6^ cells/mL each injection), numbers that were safe and showed therapeutic benefit. Nevertheless, the administration of an excessive number of BM-MNC has resulted in adverse effects in animal models [20]. Therefore, dose studies will be critical to optimizing the administration of stem cell populations in CLTI.

On the other hand, the severity of ischemia in patients that are candidate for cellular treatment is a critical aspect to consider in clinical trials. In our study, the participants were in Rutherford’s stages 3–5, and responded effectively to the cellular treatment. This finding agrees with other studies, such as Walter et al., who reported that individuals with Rutherford’s stages 4–5 did respond to cellular treatment, but those with stage 6 did not [13].

We designed our study primarily as a pilot study and proof of concept, and even though the sample size was small, one of the limitations of this study, we saw a significant clinical improvement in the auto-BM-MNC and allo-WJ-MSC-treated participants compared with the placebo group. Nevertheless, according to our research, allo-WJ-MSCs can rival auto-BM-MNC because of less invasive extraction approaches. Therefore, it is recommended to do a multi-centric randomized phase II clinical trial, which includes a large number of subjects from different geographic places, to gather more information about auto-BM-MNC and allo-WJ-MSC effectiveness. Nevertheless, our results provide new knowledge about the safety and efficacy of auto-BM-MNC and allo-WJ-MSC in diabetic patients with chronic limb-threatening ischemia.

Finally, stem cell treatment may sometimes encounter ethical obstacles or biological restrictions. Thus, in recent years, it has been thought that the released secretomes, composed of biologically active molecules (growth factors, cytokines and chemokines, angiogenic factors, extracellular matrix proteins, proteases, and genetic material secreted from stem cells), have revealed a considerable capacity for repair and regeneration of damaged cell membranes, or induce the secretion of surrounding tissues could reveal a new approach for the cell-free treatment [44–46].

## Conclusions

Our cumulative results indicate that auto-BM-MNC and allo-WJ-MSCs are safe and may trigger angiogenesis, restore blood circulation to ischemic sites, and promote tissue regeneration and functional recovery to reduce the severity of CLTI in diabetic patients. Nevertheless, the therapeutic benefit was more noticeable in a shorter time with allo-WJ-MSCs. Thus, our pilot is clinically relevant as it highlights the possible use allo-WJ-MSCs as a novel therapeutic approach, which could be part of the comprehensive management of CLTI in no-option patients.

### Supplementary Information

**Additional file 1. Fig S1**: Delivery route of the treatments. Representative image of auto-BM-MNC, allo-WJ-MSCs or placebo solution administration into the periadventitial layer of the arterial walls under eco-Doppler guidance at day 0.

## Data Availability

All data generated or analyzed during this study are included in this published article and its supplementary information files.

## Abbreviations

AEs: Adverse events
Allo-WJ-MSCs: Allogeneic Wharton jelly-derived mesenchymal stem cells
Auto-BM-MNC: Autologous bone marrow mononuclear cells
CLTI: Chronic limb-threatening ischemia
EDTA: Ethylenediamine tetraacetic acid
EC: Endothelial cells
EPCs: Endothelial progenitor cells
FOSCAL: Fundación Oftalmológica de Santander
GMP: Good Manufacturing Practice
hD-PRP: Human derivatives from platelet-rich plasma
IA: Intra-arterial
IV: Intravenous
MEM: Minimum Essential Medium Eagle
MSCs: Mesenchymal stem cells
PWD: Pain-free walking distance
PAD: Peripheral artery disease
UCB-MSCs: Umbilical cord blood-derived MSCs
VAS: Visual analog scale
