# Supplementary material for: A novel therapeutic management for diabetes patients with chronic limb-threatening ischemia: comparison of autologous bone marrow mononuclear cells versus allogenic Wharton jelly-derived mesenchymal stem cells
Source: Stem Cell Res Ther. 2023 Aug 25;14:221. doi: 10.1186/s13287-023-03427-z (PMC10464344; doi:10.1186/s13287-023-03427-z)
Supplement: Supplementary file 1 — Additional file 1. Fig S1: Delivery route of the treatments. Representative image of auto-BM-MNC, allo-WJ-MSCs or placebo solution administration into the periadventitial layer of the arterial walls under eco-Doppler guidance at day 0. [file 13287_2023_3427_MOESM1_ESM.docx]

**Supplementary figure 1. Delivery route of the treatments.** Representative image of auto-BM-MNC, allo-WJ-MSCs or placebo solution administration into the periadventitial layer of the arterial walls under eco-Doppler guidance at day 0.
